# Improved Left Ventricular Aneurysm Repair with Cell- and Cytokine-Seeded Collagen Patches

**DOI:** 10.1155/2018/4717802

**Published:** 2018-02-13

**Authors:** Hui Qu, Bao-dong Xie, Jian Wu, Bo Lv, Jun-bo Chuai, Jian-zhong Li, Jun Cai, Hua Wu, Shu-lin Jiang, Xiao-ping Leng, Kai Kang

**Affiliations:** ^1^Department of Pediatrics, The Second Affiliated Hospital of Harbin Medical University, Harbin, China; ^2^Department of Cardiovascular Surgery, The Second Affiliated Hospital of Harbin Medical University, Key Laboratory of Education Ministry for Myocardial Ischemia, Harbin, China; ^3^Department of Cardiology, The Second Affiliated Hospital of Harbin Medical University, Harbin, China; ^4^Department of Ultrasound, The Second Affiliated Hospital of Harbin Medical University, Harbin, China

## Abstract

**Background:**

Engineered heart tissues (EHTs) present a promising alternative to current materials for surgical ventricular restoration (SVR); however, the clinical application remains limited by inadequate vascularization postimplantation. Moreover, a suitable and economic animal model for primary screening is another important issue.

**Methods:**

Recently, we used 1-ethyl-3-(3-dimethylaminopropyl)carbodiimide hydrochloride chemistry (EDC) to initiate a strengthened, cytokine-conjugated collagenous platform with a controlled degradation speed. In vitro, the biomaterial exhibited an enhanced mechanical strength maintaining a porous ultrastructure, and the constant release of cytokines promoted the proliferation of seeded human mesenchymal stem cells (hMSCs). In vivo, with the hMSC-seeded, cytokine-immobilized patch (MSCs + GF patch), we performed modified SVR for rats with left ventricular aneurysm postmyocardial infarction (MI). Overall, the rats that underwent modified SVR lost less blood and had lower mortality. After 4 weeks, the rats repaired with this cell-seeded, cytokine-immobilized patch presented preserved cardiac function, beneficial morphology, enhanced cell infiltration, and functional vessel formation compared with the cytokine-free (MSC patch), cell-free (GF patch), or blank controls (EDC patch). Furthermore, the degradable period of the collagen patch in vivo extended up to 3 months after EDC treatment.

**Conclusions:**

EDC may substantially modify collagen scaffold and provide a promising and practical biomaterial for SVR.

## 1. Introduction

Currently, acute myocardial infarction (AMI) remains a leading “killer” in humans [1]. As a result of the successive adverse remodelling, even individuals who survive a lethal attack remain at risk of ventricular aneurismal formation and functional failure. Surgical ventricular restoration (SVR) rapidly normalizes the size and shape of the cardiac chamber and reverses the heart function; however, SVR fails to maintain the improvements in a long-term period [2, 3] because of the recurrent dilatation of the ventricle [3]. The repairing patch currently used may be partially responsible for the problem. The current materials for SVR are typically stiff and synthetic, which render the patch and the adjacent regions scarred and nonelastic, thus resulting in chronic stresses and contributing to ventricular redilatation and dysfunction. In contrast, biodegradable scaffolds may produce compliant tissues that heal without scarring [4]. As such, engineered heart tissues (EHTs) have attracted broad attention for their potentials to grow, repair, and regenerate, and various EHTs have been developed to improve the deficiency of the current material [5, 6].

Scaffold is one essential component of EHTs. In general, the proper scaffolds can mimic native extracellular matrix (ECM) to facilitate cell homing and metabolism, degrade at a desirable rate to enable newly formed tissue to take over the structural integrity and mechanical load, and be mechanically strong enough to sustain the pressure of the ventricle. More importantly, as a cell carrier, the biomaterial must be “vascularized” or “easy to be vascularized” to supply the seeded or recruited cells with sufficient necessities (e.g., oxygen and nutrients) or remove waste after implantation. As well documented, without a rapid formation of interior, mature vasculature between engraftment and host tissue, the viability of implanted tissue may be clearly affected or ultimately lost as a result of the insufficiency of oxygen and/or nutrients [7, 8]. To break through the barrier, a wide range of scaffolds [9, 10], cell sources [11, 12], methods, and techniques [13–15] have been tested or developed. For example, Fidkowski et al. [16] and Radisic et al. [17] created tissue engineering scaffolds with microchannels to mimic vasculature and facilitate mass transport; Sasagawa et al. [18] and Sekine et al. [12] initiated the prevascular networks by sandwiching endothelial cells (ECs) between cardiac cell sheets. By fusing vascular endothelial growth factor (VEGF) onto collagen matrix with the collagen-binding domain (CBD), Gao et al. [19] established a cell platform with proangiogenetic cytokine and accelerated the vascularization of biomaterial in vivo. However, to date, the optimal scaffold and strategy to induce vasculogenesis in scaffolds have remained uncertain.

Collagen is a natural component of cardiac ECM. Porous collagen sponge has a desirable ultrastructure, biocompatibility, and safety and may be commercially available at an economic cost. However, for SVR application, it lacks strength and rapidly degrades, which may lead to the instant or delayed rupture of the left ventricle [20]. Recently, Lorain et al. exploited a method to modify collagen scaffold. Using 1-ethyl-3-(3-dimethylaminopropyl)carbodiimide chemistry (EDC), they covalently immobilized angiogenetic cytokines into collagen patch and induced H5V EC proliferation in vitro and EC infiltration in the chorioallantoic membrane assay [21]. In addition, the strength of collagen scaffolds was substantially enhanced with limited structural change. This platform, which consists of collagen sponge tethering VEGF and cell-free, was successfully used to repair defects of the right ventricular outflow tract (RVOT) of rats [22]. In this study, we immobilized another cytokine, platelet-derived growth factor (PDGF), into the scaffolds and seeded with human mesenchymal stem cells (hMSCs). With this cytokine-conjugated and cell-seeded biomaterial, we repaired the left ventricle in a modified SVR model. We aimed to demonstrate that this modified biomaterial may be used for SVR to restore the cardiac function of rats with ventricular aneurysm.

Another point of this article is the modification of the animal model. To date, the Dor procedure has continued to be the mainstream therapy for left ventricular aneurysm, which is associated with a transmural resection of scar, endocardial circular suture, and patch implantation. After surgery, the patches employed become a part of the left ventricle and withstand the high ventricular pressure. The SVR models were initially established in large animals [23, 24] with the help of a professional technician and equipment. However, in terms of the primary screening of EHTs, these large animal models are too expensive and inconvenient. The rat is a common target of animal research, and early SVR models in rats were only a linear or circular plication of the aneurismal ventricular wall with or without epicardial patch implantation [25, 26]. These models did not imitate the Dor procedure and could not accurately testify the characteristics of EHTs. In 2003, Matsubayashi et al. [4] initially repeated the Dor procedure in a rat model. Through a median sternotomy, the transmural scar of the left ventricle was resected, and a bioengineered patch was implanted into the heart without cardiopulmonary bypass (CPB). The model mimicked the Dor procedure more properly than before with the exception of the higher surgical mortality (8/35), which was attributed to the excessive blood loss and other side injuries caused by the operation. In this study, we modified the animal model by changing the approach for SVR from the midsternal line to the 4th left anterolateral intercostal space, a different rib space from the 1st thoracotomy, which is performed to generate myocardial infarction through the 3rd rid space. We evaluated whether the modification may minimize the injury to weak rats, decrease the mortality of SVR, and provide an economic and effective small animal model for the primary screening of EHTs.

Briefly, the aim of the study was to evaluate the effectiveness of the following two modifications. The first issue is whether the approach change decreased the rat mortality of SVR. The second issue is whether this cytokine-conjugated, hMSC-seeded collagen patch may be used as proangiogenic biomaterial for SVR. Here, hMSCs seeded on the patch were used as a strategy to promote angiogenesis, and future studies will determine the optimal cocultured cell source, particularly those with potential to contract, to preserve cardiac function post-SVR.

## 2. Methods and Materials

### 2.1. In Vitro

#### 2.1.1. Preparation of Collagen Scaffold and Release Curve Examination

The collagen scaffold was prepared as previously described [21]. Briefly, a collagen sponge (shQISHENG) sheet was trimmed into uniform scaffold (diameter: 7 mm, thickness: 2 mm) with a metal borer and immersed in a sterile phosphate buffered saline (PBS) of 1-ethyl-3-(3-dimethylaminopropyl)carbodiimide HCl (EDC; Sigma, E7750, 24 mg/ml in PBS) and N-hydroxysulfosuccinimide (Sulfo-NHS; Pierce Chemicals, 24,510, 60 mg/ml in PBS). The activation step lasted for 60 min at room temperature; the scaffolds in the GF group were then sequentially immersed in a mixed solution of VEGF (Peprotech, 100–20, 1 *μ*g/ml in PBS) and PDGF (R&D, 120-HD, 1 *μ*g/ml in PBS) for 2 h for cytokine conjugation, whereas the scaffolds in the EDC group were only preserved in PBS. Finally, the GF scaffolds were washed 8 times (5 minutes each time) in fresh PBS to remove unreacted components and maintained in PBS. Collagen scaffolds without treatment were immersed in PBS (PBS group) as a control.

The amount of growth factors in the scaffolds was quantified by subtracting the amount that remained in the reaction solution and released into the washing PBS from the initial reaction amount (the initial amounts of VEGF and PDGF were 150 ng, resp.), which was measured with the commercially available ELISA kits as described by the manufacturer. To further differentiate the covalently conjugated cytokines, the level of physical absorption of cytokines was identified using the collagen scaffold without EDC treatment at the same circumstance.

Moreover, the released amounts of cytokines were measured at 1 d, 3 d, 7 d, 14 d, and 28 d, respectively, and plotted to form the release curve of the construct (*n* = 5 samples/group).

#### 2.1.2. Tensile Test and Structural Examination

The mechanical properties were evaluated using an ElectroForce 5200 BioDynamic Test Instrument (Bose, Eden Prairie, USA) with a 22 N load cell at a constant speed of 1.0 mm/min and a gauge length of 7 mm (*n* = 5/group). In this part, collagen scaffold without treatment was defined as the control (PBS group) to compare with the scaffold crosslinked with EDC (EDC group) and the scaffold immobilized with growth factors (GF-group) (*n* = 5/group). All scaffolds were cut into rectangle stripes (length: 2 cm, width: 1 cm, and thickness: 2 mm) and functionalized as previously described. Prior to tensile testing, the sponge was briefly dabbed on tissue paper to remove excess PBS and measured with a calliper for its width and thickness. Sponges were pulled until completely broken.

For ultrastructural examination, the scaffolds of each group (*n* = 5/group) were characterized under a scanning electron microscope (Hitachi S-3400N, Japan) using an image analysis system (NIS-Elements BR 3.0, Nikon, Japan). The average diameter of the pores was determined with the system, and at least 40 random pores were assessed.

The porosity of the scaffold was measured through liquid displacement as previously described [27]. Briefly, each group of scaffolds (*n* = 5/group) was lyophilized in a chamber at −80°C. The sample sponge was subsequently immersed in a graduated cylinder that contained a known volume (V_1_) of ethanol, and a series of brief evacuation-repressurization cycles were conducted until no air bubbles were observed emerging from the foam. The total volume of ethanol and the ethanol-impregnated sponge were recorded as V_2_. Finally, the ethanol-impregnated sponge was removed from the cylinder, and the residual ethanol volume was recorded as V_3_. Thus, the total volume of the sponge could be expressed as (V_2_ − V_1_) + (V_1_ − V_3_) = V_2_ − V_3_. The quantity (V_1_ − V_3_) was equal to the volume of the ethanol held in the sponge, which also represented the void volume of the scaffold. Thus, the porosity of the sponge could be determined with the following formula: porosity (%) = (V_1_ − V_3_)/(V_2_ − V_3_) × 100%.

#### 2.1.3. Cell Isolation, Cultivation, Identification, and Patch Seeding

hMSCs were isolated from the sternums of patients (with simple congenital heart disease and aged 3–10 years old) who underwent cardiac surgery, and an informed written consent was provided by each child's guardian prior to surgery. All experiments were performed under the permit of the ethic committee in the Second Affiliated Hospital of Harbin Medical University (ethic number 2014-010).

Specially, the mononuclear cells of human bone marrow were isolated via centrifugation with a Ficoll-Paque gradient (1.077 g = mL density; GE Healthcare, Kretztechnik, Zipf, Austria). The separated cells were plated in flasks and further purified by discarding the nonadherent cells after a 48 h culture with Iscove modified Dulbecco medium (IMDM; which contained 10% foetal bovine serum and antibiotics) in an incubator (37°C, 5% CO_2_.). The cells were subcultured while the confluence reached 70 to 80%, and the passage 3 cells were characterized by flow cytometry (FACS Calibur, Becton Dickinson, USA) using antibodies against CD90, CD105, CD45, and CD34.

The cell seeding procedure was the same as previously described [21]. Briefly, the scaffolds were incubated for 30 min in culture medium, dried on autoclaved kimwipes, and transferred into a 24-well plate. The hMSCs were trypsinized, centrifuged into a pellet in a desired number (0.5 × 10^6^ cells/scaffold for cell proliferation evaluation and 1.0 × 10^6^ cells/scaffold for other tests), and resuspended in the volume of medium corresponding to 10 *μ*l per scaffold. The cell resuspensions were evenly seeded into the surface of the scaffold and incubated for an additional 40 min (37°C, 5% CO_2_) to enable attachment, followed by the addition of 1 ml fresh medium. The samples were incubated for 3 days prior to patch implantation.

#### 2.1.4. Cell Proliferation Measurement

The proliferative effect of the patch was qualitatively or quantitatively evaluated by cell counting in an HE-stained slice or CCK_8_ assay, respectively. Briefly, constructs were prepared as previously described and underwent HE staining on day 2 and day 4 after 24 h fixation in 10% formalin. Then, under a light microscope, the cell number of each slide was calculated by two independent observers in a blind manner. The numbers of each group at each time point were averaged and plotted to the culturing time to obtain the growth curve of each group. For the examination, 5 slides per group (*n* = 5/group timepoint) were stained and 5 high-power fields (100x) were randomly selected at each time point.

In addition, the CCK_8_ assay (Dojindo CK04, Japan) was performed to quantitatively measure the cell number on the scaffolds after 4-day and 7-day cultivation, according to the manufacturer's instruction. Briefly, constructs (*n* = 5/group timepoint) were transferred into a 24-well plate and incubated with freshly prepared CCK_8_ labelling solution (10 mg/ml, 500 *μ*l/scaffold) at 37°C/5% CO_2_ for 4 h. The calibration curve consisted of a blank scaffold (no cells) and a known number of cells were seeded into the scaffolds (0.2 × 10^6^, 0.4 × 10^6^, 0.8 × 10^6^, or 1.6 × 10^6^). The supernatants of both the constructs and the standards were removed to a 96-well, and the absorbance of each supernatant was measured at 450 nm using a plate reader (3 replicates/sample) (Bio-tech Inc., BOX998, USA). The amount of formazan dye formed, as indicated by the absorbance, was proportional to the number of viable metabolizing cells.

### 2.2. In Vivo

#### 2.2.1. Experimental Animals

Adult Sprague Dawley female rats that weighed 250–280 g were used in our study. All animal procedures were in accordance with the Guide for the Care and Use of Laboratory Animals (NIH Publication number 85-23, revised 1996) and were approved by the Animal Care and Use Committee of the Second Affiliated Hospital of Harbin Medical University. To avoid immunorejection, all animals were intraperitoneally administered with cyclosporine A (5 mg/kg, Novartis) each day from 3 days before patch implantation to the end of the investigation.

#### 2.2.2. Surgical Procedure

The animal procedure was performed as previously described by our group, with an approach alteration [21, 28]. Briefly, all rats underwent left anterior descending artery (LAD) ligation to generate transmural myocardial infarction (MI) through the 3rd left anterolateral intercostal space. After two weeks, an echo examination was performed to screen the rats by checking the infarct area. Only the animals with a kinetic infarct area greater than 25%, but less than 35%, of the left ventricular free wall were enrolled in this study. These rats (with a uniform scar size) were randomly assigned to the following two groups and underwent SVR: the control group (through a midsternal approach) (*n* = 20) and experiment group (through a left anterolateral 4th intercostal space) (*n* = 49). The rats assigned to the experiment group were then randomly subdivided into four groups, including EDC = collagen scaffold treated with EDC chemistry alone (*n* = 15); GF = collagen scaffold treated with EDC chemistry and conjugated with cytokines (*n* = 12); MSC = collagen scaffold modified with EDC chemistry and seeded with hMSCs (*n* = 11); and MSC + GF = collagen scaffold crosslinked with EDC, immobilized with cytokines, and seeded with hMSCs (*n* = 11). For the rats in the experiment group, the procedures were performed through a left anterolateral 4th intercostal approach. Briefly, through the presetting approach, a purse-string suture was placed along the infarct area and snored down with a tourniquet to plicate the neck of the aneurysm. The transmural scar was resected, and the corresponding defect was repaired with individual patches. (For the MSC and MSC + GF patch, the cell-seeded surface was placed on the endocardial side). The tourniquet was carefully released, and the purse-string stitch was removed after a continuous over-and-over stitch with 7–0 polypropylene. The chest was closed, and the rat recovered under careful monitoring. Similarly, the rats in the control group underwent the same procedure with the exception of the midsternal incision. For surgery, all gauze for each rat must be weighed, and the bloodstained gauze was collected in a closed container. The total gauze was weighed again, and the surgical blood loss was identified by the difference in the total gauze weight.

All surgical procedures were performed by the same experienced cardiac surgeon. To ensure that the animals underwent the transmural repairing, four rats (*n* = 1/group) were randomly sacrificed to directly observe 3 days after patch implantation.

#### 2.2.3. Degradability of EDC-Modified Scaffold

To detect the degradation of biomaterials in vivo, the observational schedule of three rats (randomly selected) in the EDC group (*n* = 2) was prolonged to three months. At the end of the study, the rats were sacrificed, and the hearts were removed. The implanted patches were photographed, and the regenerative tissues were fixed, embedded, and underwent Masson Trichrome's staining to observe the degradation of the collagen scaffold.

#### 2.2.4. Assessment of Cardiac Function

The left ventricular (LV) function was evaluated via echocardiography (Sequoia C256 System, Siemens Medical; 15 MHz linear array transducer) before MI (preligation baseline), before patch implantation (day 0), day 7 and day 28 after patch implantation in all animals. M-mode and 2-D images were obtained in the parasternal short axis at the level of the papillary muscles. The measurement was performed by an experienced examiner in a single blind fashion, and 5 consecutive cardiac cycles were recorded and averaged. The LV internal diastolic dimension (LVIdD) and internal systolic dimension (LVIsD) were determined in M-mode imaging, and the fractional shortening (FS%) of the LV was calculated with the following formula: FS% = [(LVIdD-LVIsD)/LVEdD)] × 100%. Accordingly, the cross-sectional areas of the left ventricle at the end of systole (LVESA) and diastole (LVEDA) were measured with built-in software in 2-D imaging, and the fractional change area (FCA%) was calculated with the following formula: FCA% = [(LVEDA-LVESA)/LVEDA)] × 100%.

After the last echocardiography, the right carotid artery of each animal was exposed, and a 2F pressure transducer (Millar Instruments, USA) was cannulated into the left ventricle through it (*n* = 10/group). The load-dependent parameters were measured at a steady state. The abdominal cavity was subsequently opened, and the inferior vena cava was transiently occluded to record the load-independent parameters. All measurements were recorded with a PowerLab Chart 5 system (AD Instruments) and analysed with Millar PVAN 3.3 software (Millar Instruments, USA).

#### 2.2.5. Morphological Evaluation

At 28 days after patch implantation, all animals (with the exception of subjects in the degradability experiment) were euthanatized by a 10% kalium chloride (KCL) intracardiac injection. For each rat, the heart was subsequently removed, and an intraventricular balloon was filled to 30 mmHg of pressure for vertical photography at a scheduled distance (72 cm). The patch areas of all groups (*n* = 10/group) were measured using ImageJ software and calculated as previously described [29].

Next, the samples were fixed with 4% paraformaldehyde for 24 h, followed by sucrose of different concentrations for different periods (10% for 1 h, 20% for 1 h, and 30% for 24 h). The hearts were subsequently cut in half along the centre of the patch and snap frozen in the molds filled with OCT compound. The samples (*n* = 10/group) were sliced into 5 *μ*m thick sections and stained for Masson's Trichrome. The patch thickness was quantified using computerized planimetry (ImageJ software) as previously described [29].

#### 2.2.6. Measurement of Functional Vessels

To evaluate the functional vessel formation of cardiac tissue, a 5 *μ*m-thick cytosection was sliced as previously described. The slices from each group of animals (*n* = 10/group) were then immunofluorescently stained with antibody against *α*-smooth muscle actin (SMA, Santa Cruz, SC-6956) according to the manufacturer's instruction and observed under a fluorescence microscope (Nikon Eclipse TE200, Japan). The captured image was converted into black and white mode, and the cross-sectional area of each vessel in the image was illustrated (ImageJ software). In this case, functional vessels were defined as vessels with an area greater than 100 *μ*m^2^. The vessel number that conformed to the criteria was counted and normalized to the tissue area to form the mature arteriole density. Moreover, the total cross-sectional area of these vessels and their fraction to the tissue area were calculated to accurately evaluate the tissue perfusion.

### 2.3. Statistical Analyses

Statistical analysis was performed with GraphPad Prism5 software (San Diego, Calif). Data were all expressed as the mean ± standard deviation (SD). A mortality comparison through the different approaches was performed with fisher's exact test (chi-square test), and the corresponding blood loss was compared with *t*-test. All comparisons among multiple groups were conducted via one-way ANOVA with the exception of the cell counting and echocardiography, which were determined with two-way ANOVA. If the F ratio was significant, posttests were conducted using the Turkey test or Bonferroni test, respectively. *P* < 0.05 was considered statistically significant.

## 3. Results

### 3.1. EDC Treatment Enhanced the Mechanical Property of Collagen Scaffold without Changing the Ultrastructure

**Figure 1:**
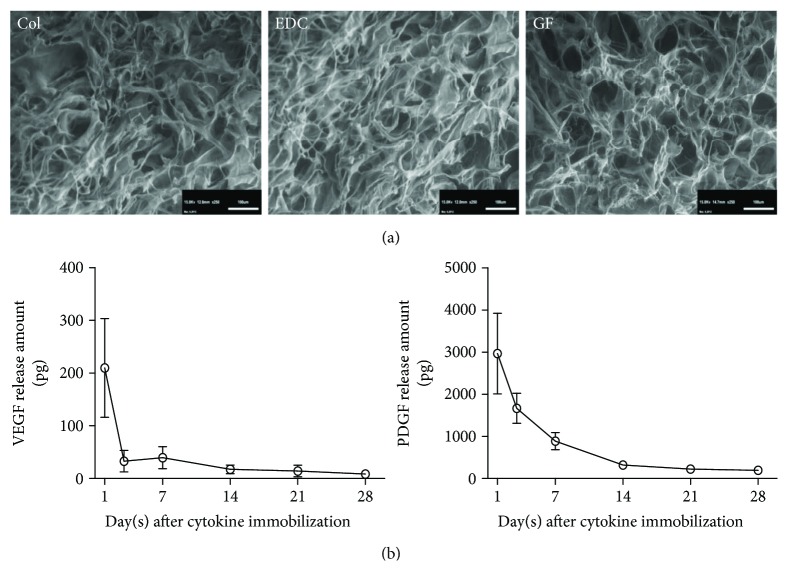
Characterization of the scaffolds. (a) Scan electron microscopy (SEM 250x) showed that the collagen sponge maintained a similar porosity and porous size after EDC chemistry and cytokine immobilization. (b) Enzyme-linked immunosorbent assay (ELISA) demonstrated that both vascular endothelial growth factor (VEGF) and platelet-derived growth factor (PDGF) immobilized in scaffolds were released in a constant and controlled modes.

In general, the EDC treatment substantially enhanced the stiffness and strength of the collagen scaffolds, and the following cytokine immobilization did not adversely affect their usefulness for SVR. Specially, compared with the PBS patch, the Young's modulus (a measure of stiffness) of the EDC scaffolds was significantly strengthened (EDC versus PBS, ^∗∗^ *p* < 0.01), and the cytokine immobilization was affected, but did not significantly decrease the indicator (GF versus PBS, ^∗∗^ *p* < 0.01, EDC versus GF, *p* > 0.05). Similarly, the ultimate tensile strength (the maximum tensile stress to withstand stretching) of the EDC or GF patch was substantially higher than that of the PBS patch (EDC, GF versus PBS, ^∗∗^ *p* < 0.01), although with a significant degradation for cytokine conjugation (EDC versus GF, ^∗^ *p* < 0.05) (Table 1). Fortunately, the mechanical improvement did not change the porous structure of collagen (Figure 1(a)), and the average pore size and porosity remained comparable among the three groups (EDC, GF versus PBS, *p* > 0.05) (Table 1).

### 3.2. Immobilized Growth Factors Were Constantly Released and Facilitated Cell Proliferation In Vitro

The release kinetics of the cytokines in the scaffold are illustrated in Figure 1(b). Both cytokines released relatively faster in the first three days and then exhibited a controlled release mode during the 4-week period. At the end of the experiment, the total amounts of released VEGF and PDGF were 322 pg and 4980.2 pg, respectively, and substantial amounts of growth factors were immobilized into the scaffolds.

Morphologically, the cultured cells displayed a homogeneous spindle-shaped population. The flow cytometry analysis showed that over 95% of hMSCs expressed CD90, and CD105, whereas less than 2% of the cell population expressed leukocyte common antigens, CD45, or the hematopoietic lineage markers CD34 (*n* = 5/group) (Fig. Supp 1).

**Figure 2:**
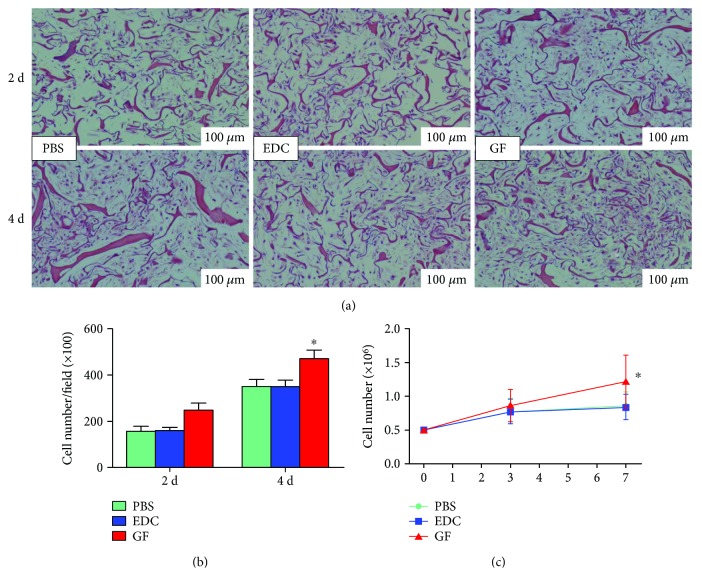
Proliferation of hMSCs in scaffolds in vitro. (a) Representative micrographs (100x) of HE staining for human mesenchymal stem cells (hMSCs) in PBS-treated scaffold (PBS), crosslinker (EDC/Sulfo-NHS)-treated scaffold, and scaffold immobilized with VEGF + PDGF (GF) after 2-day and 4-day cultivation. (b) The bar graph shows that cells increased in all scaffolds over time and grew most quickly in cytokine-immobilized scaffold (GF) (4D: ^∗^ *p* < 0.05 GF versus PBS, EDC). No difference was identified between EDC and PBS scaffolds. (c) The CCK_8_ assay performed on day 3 and day 7 after cell seeding illustrated similar results quantitatively (7 D, ^∗^ *p* < 0.05 GF versus PBS, EDC).

We further identified a large quantity of CD105-positive cells in collagen patches after 4-day planting (Fig. Supp 2). Detailedly, the EDC treatment did not affect the viability of seeded hMSCs because the cell number in the EDC patch did not significantly differ from that in the PBS patch throughout the experiment. Comparatively, the cytokine conjugation exerted a markedly beneficial effect on cell proliferation (Figures 2(a)–2(c)) because the cell number in the GF patch was consistently the largest among the three groups at 2 and 4 days after cell seeding (D_4_: GF versus EDC, PBS ^∗^ *p* < 0.05). Moreover, our CCK_8_ assay performed at 3 and 7 days after cell seeding further quantitatively confirmed the results (GF versus EDC, PBS ^∗^ *p* < 0.05) (Figure 2(c)).

### 3.3. Modification of Approach for SVR Decreased Surgical Blood Loss and Mortality

**Figure 3:**
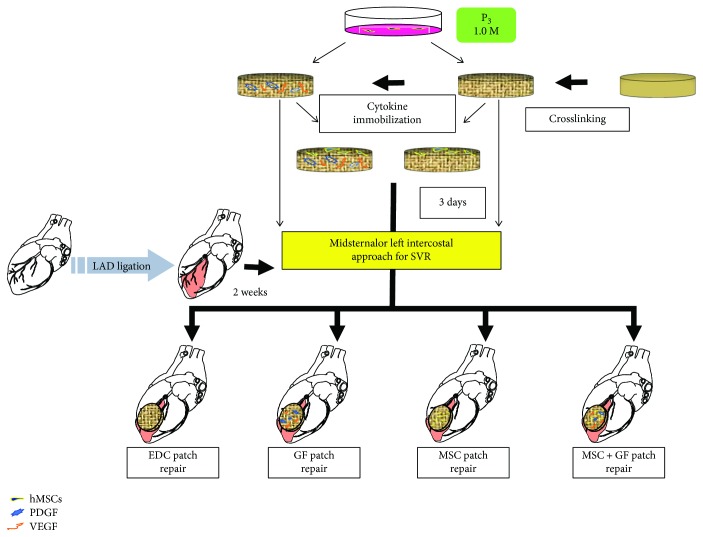
Experimental design. Schematic representation of patch preparation, MI, SVR, and patch implantation, with experimental test groups shown.

**Figure 4:**
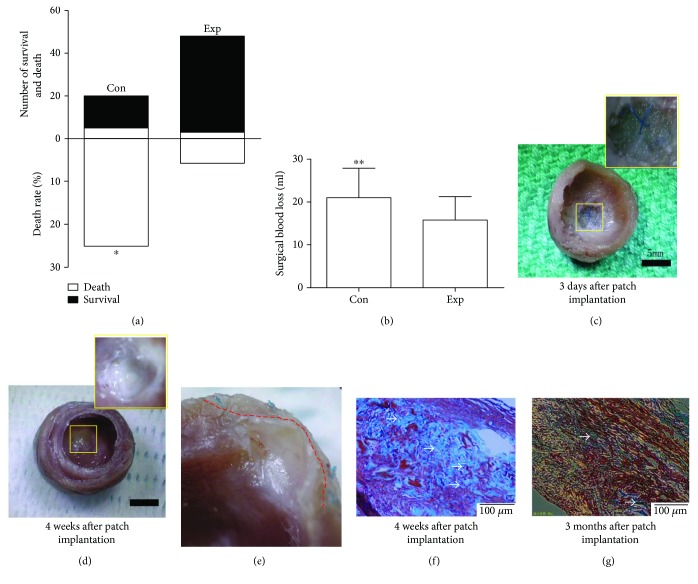
Modification of animal model and patch degradation in vivo. (a) and (b) Surgical mortality and blood loss significantly decreased by changing the approach of SVR from the midsternum (control group) to the 4th left anterolateral intercostal space (experimental group). (mortality: ^∗^ *p* < 0.05 Exp versus Con; blood loss: ^∗∗^ *p* < 0.01 Exp versus Con). (c)–(e) 3 days after patch implantation, one rat in each group was sacrificed. Interventricular image identified the full-thickness replacement of the left ventricle by the patch. Four weeks after implantation, the patch had been endothelialized and became a part of the left ventricle. The dotted line (red) indicates the approximate edge of neoendocardium and collagen patch. (f) and (g) Representative micrographs of Masson Trichrome's staining (100x) of crosslinked patch. At 4 weeks after implantation, the collagenous networks in the core of the patch could be clearly identified; however, most of them degraded, and limited residual debris remained after 3 months (white arrow indicated).

**Figure 5:**
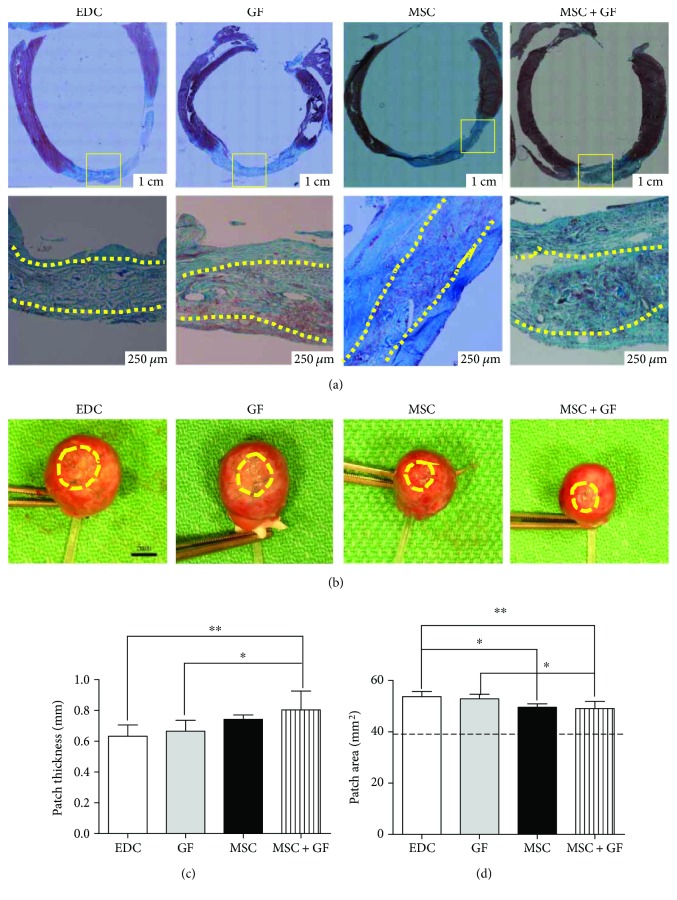
Morphologic and recellularized analysis of patch 28 days after implantation in vivo. (a) Representative images of heart slices stained with Masson's Trichrome to indicate the patch thickness and patch recellularization (100x). Thicker neoendocardium and denser cellular distribution in scaffold were identified in the GF patch, MSC patch, and MSC + GF patch compared with the EDC control (dotted yellow line separated collagen patch from newly formed endocardium and epicardium). (b) Representative images of rat hearts showed the outer border of the patches (yellow broken line indicated). (c) Compared with the EDC patch, cytokine conjugation or hMSC seeding induced an insignificant increase in thickness. The combination of cytokines and cells resulted in the thickest patch (^∗^ *p* < 0.05 MSC + GF versus GF; ^∗∗^ *p* < 0.01 MSC + GF versus EDC); however, there was no significant difference among the EDC, GF, and MSC patches or between the MSC and MSC + GF patches. (d) Comparatively, hMSC seeding resisted patch expansion more effectively than cytokine immobilization because the patch area in both cell seeding groups was significantly smaller than its cell-free control (^∗^ *p* < 0.05 MSC versus EDC; ^∗^ *p* < 0.05 MSC + GF versus GF). Eventually, the combination of cells and cytokines led to the smallest patch (^∗∗^ *p* < 0.01 MSC + GF versus EDC). Black broken line indicates patch area at the time of implantation.

The animal study was designed as shown in Figure 3. Seventy-six rats underwent left artery descending branch (LAD) ligation, and 3 rats died of the causal myocardial infarction (MI). After 2 weeks, 4 rats were ruled out by echo screening because of a too large or small akinetic infarct area, and the remaining 69 rats were randomly assigned to the control (through midsternal approach, *n* = 20) and experiment (through left anterolateral 4th intercostal space, *n* = 49) groups for SVR. As shown in Figure 4(b), the blood loss in the experimental group (SVR through intercostals) was substantially lower than that in the control group (SVR through sternum) (Exp versus Con, ^∗∗^ *p* < 0.01). Accordingly, the mortality also significantly decreased from 25% (5/20, our data) or 23% (8/35, Matsubayashi's data) to 6.12% (3/49) (Exp versus Con, ^∗^ *p* < 0.05) (Figure 4(a)). Furthermore, all subjects sacrificed on day 3 after patch implantation showed the full-thickness wall replacement (Figures 4(c)–4(e)). After 4 weeks, all engrafts were endothelialized and became a part of the left ventricle (Figure 5(a)).

Furthermore, the EDC treatment endowed the collagen sponge with the resistance to enzymatic degradation, which used to be an important concern for collagen as a scaffold. As our in vivo experiment demonstrated, the collagen patch could be clearly observed with more or less cell and tissue infiltration (Figure 4(f)) 4 weeks after implantation. After 3 months, most collagenous network disappeared, and limited residual debris could be detected (Figure 4(g)).

### 3.4. Cytokine Immobilization Enhanced Therapeutic Effect of Seeding Cells on Cardiac Function

**Figure 6:**
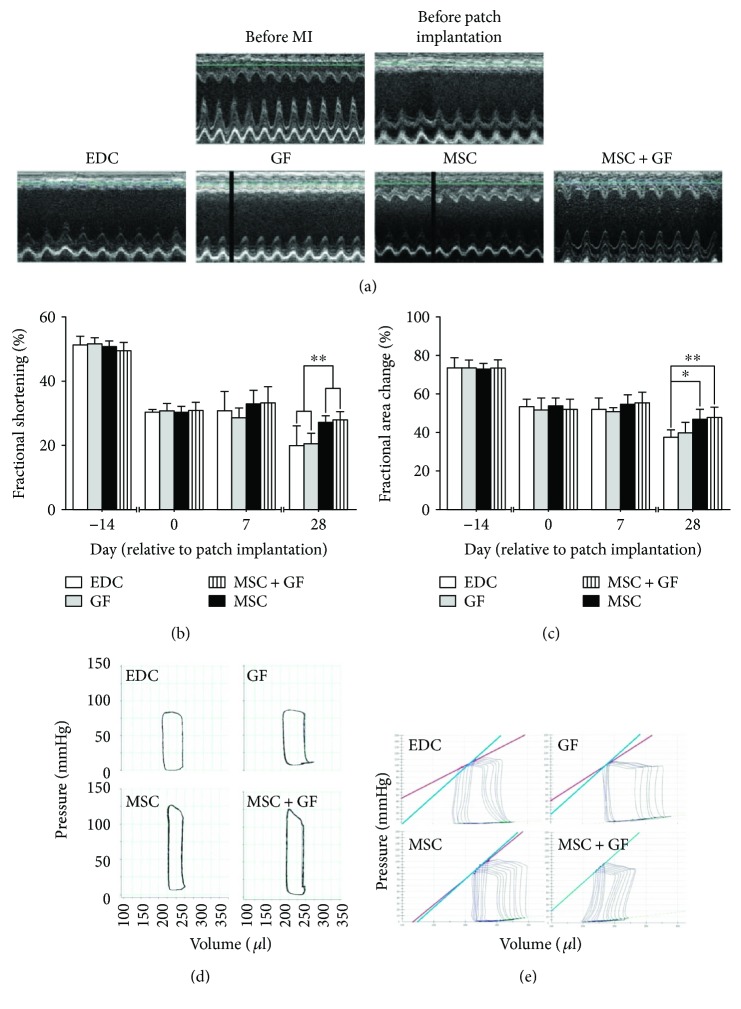
Cardiac function 28 days after patch implantation in vivo. (a) Representative M mode echocardiographic images before myocardial infarction, before patch implantation (D_0_), 7 days (D_7_) and 28 days (D_28_) after patch implantation. (b) Fractional shortening (FS%) and (c) fractional area change (FAC%) substantially decreased in all groups at a similar speed to a comparable level before patch implantation (Day_0_). However, after patch implantation, the cardiac function depressed relatively slower in both cell seeding groups, which presented as the more properly preserved FS% and FAC% at 28 days after patch implantation (FS%: ^∗∗^ *p* < 0.01 MSC + GF versus GF, EDC; ^∗∗^ *p* < 0.01 MSC versus GF, EDC. FCA%: ^∗∗^ *p* < 0.01 MSC + GF versus EDC; ^∗^ *p* < 0.05 MSC versus EDC). (d) and (e) Representative pressure-volume loop in a steady state or in response to vena cava occlusion. The end-systolic elastance in the MSC + GF group (blue line) was compared with that in the other three groups (red lines).

Coronary artery ligation produced significant left ventricle dilatation and stable ventricular aneurysm formation in most animals at the time of patch implantation. As a result of the echocardiographic prescreening, all parameters were virtually indistinguishable among all groups (Figures 6(b) and 6(c)), which demonstrated a similar cardiac function deterioration prior to SVR. In the following period, the cardiac function of all animals continually declined at different speeds. In general, cardiac dysfunction progressed more rapidly in rats repaired with a cell-free patch, which manifested as the dramatically decreased fractional shortening (FS%) and fractional area change (FAC%) at 28 days after patch implantation (FS%: MSC + GF versus GF, EDC, ^∗∗^ *p* < 0.01; MSC versus GF, EDC, ^∗∗^ *p* < 0.01. FCA%: MSC + GF versus EDC, ^∗∗^ *p* < 0.01; MSC versus EDC, ^∗^ *p* < 0.05). Cytokine conjugation exerted beneficial effects on cardiac function because the previously described indicators were relatively higher in both cytokine-conjugated groups than their cytokine-free controls; however, the improvement was not significant.

In the haemodynamic examination with the pressure-loop catheter, similar results were confirmed at the end of the study. As shown in Table 2, the load-dependent (ejection fraction, preload adjusted maximal power, end systolic volume, and end diastolic volume) and load-independent (maximal elastance, end systolic pressure-volume ratio, preload recruitable stroke work, and end systolic elastance) indices in both cell-seeded patch groups were substantially improved. Similarly, the immobilized cytokines exerted a positive but insignificant effect on the improvement.

### 3.5. Cytokine Immobilization and Cell Seeding Prevented Patch Expansion and Thinning

Patch thickness was assessed using heart slices (at the same level) stained with Masson's Trichrome. In general, compared with the EDC patch, the treatment with cytokine-conjugation or cell seeding alone increased the thickness of the patch; however, this increase was not significant. Moreover, the combination of cytokine and cell produced the thickest patch, with a significant increase compared with the EDC patch or GF patch (MSC + GF versus EDC, ^∗∗^ *p* < 0.01; MSC + GF versus GF, ^∗^ *p* < 0.05). However, there was no statistical significance among the EDC patch, GF patch, and MSC patch (Figure 5(c)).

For the patch area, different levels of patch dilation were identified under the pressure of the left ventricle at 28 days after patch implantation. Similarly, the EDC patch expanded the most, whereas the MSC + GF patch expanded the least, with a significant difference between them (EDC versus MSC + GF, ^∗∗^ *p* < 0.01). Comparatively, cell seeding resisted patch dilation more effectively than cytokine coupling because a significantly smaller area was observed in both cell-seeded patches than their cell-free controls (MSC versus EDC, ^∗^ *p* < 0.05; MSC + GF versus GF, ^∗^ *p* < 0.05). However, the same phenomenon was not identified between both cytokine-conjugated patches and their cytokine-free controls (GF versus EDC, *p* > 0.05; MSC + GF versus MSC, *p* > 0.05) (Figure 5(d)).

### 3.6. Covalent Immobilization of Cytokines Encouraged Cell Infiltration and Functional Vessel Formation in Scaffold

Using Masson's Trichrome staining (100x), a thicker neoendocardium was observed and more cell infiltration and repopulation were identified in the core of the collagen scaffold by cytokine conjugation, as well as cell seeding. In contrast, the EDC patch presented a thinner endothelial layer and less cellular distribution inside of the scaffold. Eventually, the combined application of cell and cytokines induced the best recellularization of biomaterial (Figure 5(a)).

**Figure 7:**
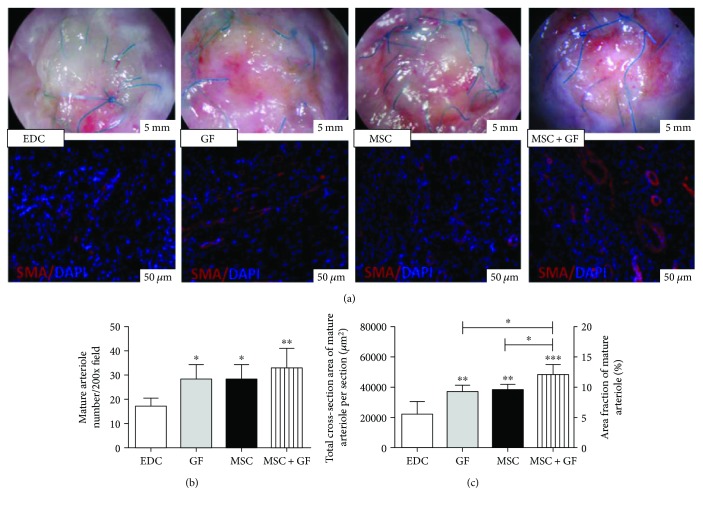
Analysis of blood perfusion in tissues peri-patch 28 days after implantation in vivo. (a) Representative gross images indicated the vasculogenesis around the patch, and representative micrographs (200x) of immunostaining for *α*-SMA (red) and DAPI (blue) identified arterioles and nuclei, respectively. (b) Density of mature arterioles (defined as vessels with a cross-sectional area greater than 100 *μ*m^2^) was significantly higher in the GF and MSC patches (^∗^ *p* < 0.05 GF versus EDC; ^∗^ *p* < 0.05 MSC versus EDC) and highest in the MSC + GF group (^∗∗^ *p* < 0.01 MSC + GF versus EDC); however, there was no significant difference among the GF, MSC, and MSC + GF groups. (c) The average total cross-section area of mature arterioles and their corresponding area fraction illustrated the individual or synergetic effects of hMSCs or cytokines on patch arteriogenesis (^∗∗^ *p* < 0.01 GF, MSC versus EDC; ^∗^ *p* < 0.05 MSC + GF versus GF, MSC; ^∗∗∗^ *p* < 0.001 MSC + GF versus EDC).

In terms of the mature arteriole formation (detected by SMA staining and calculated according to the previously described criteria), cytokine conjugation or cell seeding alone or both significantly increased the arteriole density compared with that of the EDC patch (Figure 7(c) GF, MSC versus EDC, ^∗^ *p* < 0.05; MSC-GF versus EDC, ^∗∗^ *p* < 0.01). The combined application of these two treatments resulted in the highest functional vessel density; however, it failed to have a significant advantage over the single treatment (Figure 7(b) MSC + GF versus GF, MSC, *p* > 0.05). Comparatively, another evaluation method (which focused on the vessel cross-sectional area rather than vessel number) more clearly illustrated the synergetic effect of coupled cytokines and engrafted cells on therapeutic angiogenesis (Figure 7(c) MSC + GF versus GF, MSC, ^∗^ *p* < 0.05; MSC + GF versus EDC, ^∗∗∗^ *p* < 0.001; GF, MSC versus EDC, ^∗^ *p* < 0.01).

## 4. Discussion

To date, the insufficient revascularization of biomaterial has constituted a leading challenge to the clinical translation of EHTs. In previous decades, numerous studies based on GF delivery, gene therapy, or cell-based therapy have been initiated to promote myocardial angiogenesis; however, the optimal strategy for this purpose remains uncertain. Nevertheless, one general consensus is that local and constant stimulation of proangiogenic growth factors may promote the vasculogenesis of ischaemic myocardium [30]. Furthermore, for SVR application, solid scaffolds with a specific ultrastructure, proper biocompatibility and biodegradability, and reliable mechanical property appear to be a more practical choice than other approaches.

In this work, collagen sponge was selected as the scaffold of EHTs. Collagen is an important natural component of the cardiac ECM, with weak antigenicity and excellent biocompatibility [31, 32]. It is also an FDA-approved haemostatics, and the porous 3-D structure and unique ECM protein provide an appropriate microenvironment for cell adhesion and infiltration, blood invasion, and oxygen or nutrient supply. These advantages together with the ease of access and low cost make it widely used in the tissue engineering field [33–35]. Nevertheless, in terms of the full-thickness repair of the left ventricle, a normal collagen sponge could not serve as scaffold because of its poor strength and rapid degradability in vivo [36]. To achieve this goal, necessary modification is mandatory. Thus, we modified the collagen scaffold with EDC chemistry. As shown in Table 1, by crosslinking, the mechanical properties of the collagen patch were dramatically enhanced to the level of resisting the stretch of the left ventricle. Eventually, as demonstrated by our in vivo observations, no rupture of the patch occurred in all groups until the end of the study. In this case, the beneficial effects of EDC treatment were diverse. On one side, the structure of both cross-linked patches did not compromise their mechanical improvement because the pore size and porosity of the collagen scaffolds remained similar before and after the cross-linking. Moreover, the in vivo degradable rate of collagen was markedly prolonged. As this study showed, until three months after patch implantation, collagenous residual debris was sporadically identified (Figure 4(g)), which was sufficiently long to permit new tissue formation. In addition, our proliferative test indicated that EDC had a limited toxic effect on cells seeded in the patch (Figure 2). In contrast, the immobilized cytokines by this means exhibited a controlled release mode, which promoted cell proliferation in vitro (Figure 2) and cell infiltration in vivo (Figure 5(a)). In short, the present study demonstrated that this EDC-modified collagen sponge (with or without cytokine immobilization) could be used as a scaffold to repair a transmural defect of the left ventricle.

Covalent immobilization of growth factors with EDC was another point of this study. It has been well documented that the presence of specific angiogenic cytokines postmyocardial injury may accelerate the cardiac recovery by promoting vessel formation [37, 38]. Furthermore, the functional arteriogenesis is more important than angiogenesis. Although growth factors clearly play important roles in angiogenesis and arteriogenesis, the appropriate mode for making these factors available at the desired site with the desired dosage for a desired period of time remains unclear. Given that neovasculogenesis postmyocardial injury was a site-specific, time-lasting process, the supporting growth factor should be supplied in a similar mode rather than via systemic or bonus delivery [39]. Thus, it appeared to be a favourable strategy by incorporating growth factor into biomaterial to achieve local, sustained release. As such, we initiated this platform to localize the cytokine activity, prolong receptor/ligand signalling, and, as a result, accelerate vessel formation. With respect to why VEGF and PDGF were selected as target growth factors, the reason lies in the following facts: (1) the relative high affinity to collagen by EDC chemistry. As our data indicate, the efficiencies of covalent immobilization or physical bonding were 41.6% or 2%, respectively, for VEGF and 34.1% or 3%, respectively, for PDGF, which demonstrate that substantial cytokines were chemically tethered into the scaffolds. (2) The synergistic effect on mature vessel formation. VEGF has been well established as a powerful growth factor to induce an irregular, hyperpermeable network [40], whereas PDGF could promote the maturation of developing vessels by recruiting and supporting mural cells to cover the primitive tubular vessels [41, 42]. Codelivery of the two angiogenic cytokines may markedly shorten the period of functional vessel network formation [39], which was important for transplanted cell survival and self-renewal of host tissue.

Another factor in vascularizing biomaterials was the identification of optimal seeding cells. An early cell source was typically terminally differentiated cell lines (e.g., endothelial cells, fibroblast cells, or cardiomyocytes), with the strategy to triculture these cells on scaffold simultaneously [43, 44] or sequentially [45] to mimic native cardiac tissues. Recently, with the improved understanding of angiogenesis, the proangiogenic effect of mesenchymal stem cells (MSCs) has attracted broad interests. Gaebel et al. identified that hMSCs had a protective effect on cocultured endothelial cells [25, 28], and their cardiac patch seeded with these two cell sources in a defined pattern produced enhanced vessel formation; Simpson et al. [46] indicated that both hMSCs and human embryonic stem cell-derived mesenchymal cells (hES-MCs) could enhance the neovessel formation of collagen scaffold compared to a nonviable control. Moreover, the enhancement of vascularization resulted in an improvement of the cardiac function in both trials.

This study presented similar results. Both of the hMSC-seeded groups, whether cytokine-conjugated or cytokine-free, exhibited enhanced blood perfusion and preserved cardiac function compared with its individual cell-free control. One potential explanation may be that seeded hMSCs could secrete an amount of ECM into the cardiac patch, which increased the thickness (as well as the stiffness) of the patch to withstand the stress of circulatory flow and preserved the normal geometry of the heart chamber. More importantly, it also provided an appropriate environment for cell homing to form neovessels. The conjugated cytokines augmented the process by inducing more cell recruitment and infiltration into the core of the 3-D scaffold (Figure 5(a)). The enhanced angiogenesis, particularly the arteriogenesis, subsequently contributed to the survival of transplanted cells and the viability of host progenitor cells along and remote to the patch, which accelerated the tissue regeneration and reduced the scar formation. As a result, the redilation of the left ventricle was prevented, and the heart function was preserved.

In this study, the individual angiogenic effect of delivered cytokines or hMSCs was also clearly illustrated (Figure 7). Immobilized cytokines induced similar neovessel formation to that of transplanted cells; however, they failed to significantly improve cardiac function. The addition of hMSCs alone on the patch achieved a similar protection on cardiac function irrespective of the proangiogenic inferiority to the combination of cytokines and cells. Moreover, the seemingly paradoxical phenomenon may lie in the following facts: (1) The viability of seeding cells played a predominant role in cardiac tissue engineering [47]; (2) Apart from vascularization, other mechanisms, such as paracrine effects on the host heart or transdifferentiation of transplanted cells, may also exert an influence on heart function [48].

Another point to stress here was the modification of the animal model. As previously discussed, each model required two sequential operations to first induce ventricular aneurysm and then repair it. Comparatively, the second operation was more dangerous because the surgery was performed in the rats of MI and the reoperation was often associated with increased adhesion, bleeding loss, and higher mortality, as reported by Matsubayashi et al. [4]. Thus, some measures must be taken to address the problems. The following discussion describes our experiences.

First, the LAD was ligated at the proper level to generate the appropriate infracted wall of the LV. A higher ligation may lead to a more extensive scar and worse cardiac function and, as a result, a higher mortality of rats. A lower ligation may fail to induce transmural infarction and aneurysmal formation. Our experience was to ligate the LAD approximately 2 mm under the left atrial appendage. Second, we set the time point of the second operation at two weeks after LAD ligation. If the interval time was shorter, the scarred tissue from MI was unstable and the edge between the normal and infracted area was unclear. Thus, it was difficult to judge the region of scarred tissue and correctly replace it. Definitely, an extended period after MI indicated worse cardiac function, which may convert to the higher mortality of the second operation. Finally, we implemented a series of measures to decrease the operational bleeding because it was regarded as the crucial cause of rat surgery-related death and postoperative adhesion. The measures included (1) increasing the successful rate of LAD ligation with “one-stitch” and leaving residual thread as little as possible. A practical method is to clamp the targeted portion to observe the pale area before stitching. (2) Changing the surgical approach on the second operation (from a previous median sternotomy to the 4th left anterolateral thoracotomy). Although it only appeared to be a change of incision, the clinical meanings were significant: (1) In general, surgery-related injuries, such as operative bleeding and postoperative adhesion, were more serious in median sternotomy than those in intercostal thoracotomy, which may render to the blurred image of echocardiography, the declined cardiac function, and the difficulty of sample harvesting. (2) The infarcted myocardium could be more clearly observed through the left intercostal thoracotomy, which substantially helped the operator to avoid moving the weak heart. (3) In light of the special anatomy of the rat sternum, part of the diaphragm and abdominal wall had to be dissected, and a unilateral or bilateral pleura of the rat was liable to be ruptured when a sternotomy was performed. Thus, rats that underwent sternotomy were at high risk of respiratory dysfunction because of the adverse influence on both thoracic and abdominal respiration. (4) Finally, it was more convenient when the haemodynamics were measured with a pressure-volume catheter (associated with abdominal opening and inferior vena cava occlusion) and heart samples were harvested (through the intact sternum) if the SVR was performed through the intercostal space. Fortunately, our statistical data identified the advantage of the modification (Figure 4). The blood loss of SVR through intercostals was substantially lower than that through the sternum; thus, the mortality also significantly decreased from 25% (5/20, our data) or 23% (8/35, Matsubayashi's data) to 6.12% (3/49).

### 4.1. Study Limitations

With the exception of the degradation examination, the current study comprised a short-term study that used small animals. Additional long-term studies should be performed in large animals or humans to simulate clinical conditions. With respect to seeding cells, hMSCs were used to identify their potential to vascularize biomaterials. In terms of the regeneration of native myocardium, other cell sources may be added to this “vascularized” platform to produce contractile, conductive, and self-healing heart tissue.

## 5. Conclusion

Collagen scaffolds were translated into strengthened, degradation-resistant, and cytokine-conjugated cardiac platforms for SVR with EDC. The mortality of SVR in rats was substantially decreased by the modification. The sustained release of growth factors promoted cell proliferation in vitro and functional vessel formation in vivo. The addition of hMSCs further enhanced the process, resisted patch expansion, and preserved the cardiac function. This novel EHT provided a practical and promising alterative for current SVR application.

## Figures and Tables

**Table 1:** Mechanical and structural parameters.

Group	Max tension strength (Kpa)	Young's modulus (Kpa)	Porosity (%)	Pore size (*μ*m)
PBS	18.0 ± 2.1	8.6 ± 1.0	92.3 ± 3.39	94.1 ± 6.34
EDC	38.5 ± 5.2^#∗^	19.3 ± 4.3^#^	90.5 ± 2.00	90.4 ± 5.22
GF	31.2 ± 2.9^#^	16.6 ± 0.9^#^	89.7 ± 2.10	91.4 ± 5.42

^#^ *p* < 0.01 EDC, GF versus PBS; ^∗^ *p* < 0.05, EDC versus GF.

**Table 2:** Conduction catheter data.

	EDC	GF	MSC	MSC-GF
EF (%)	24.1 ± 3.8	24.6 ± 3.6	32.5 ± 2.6^#^	33.1 ± 4.8^#^
PAMP (mWatts/*μ*l^2^)	3.62 ± 0.60	3.71 ± 0.48	4.98 ± 0.82^∗^	5.22 ± 0.93^#^
ESV (*μ*l)	288.3 ± 35.1	280.7 ± 30.9	217.6 ± 28.9^#^	208.8 ± 27.2^#^
EDV (*μ*l)	384.5 ± 45.8	371.6 ± 33.7	305.1 ± 21.6^#^	299.1 ± 21.1^#^
E*max* (mmHg/*μ*l)	1.28 ± 0.28	1.34 ± 0.27	1.73 ± 0.17^∗^	1.81 ± 0.16^#^
ESPVR (mmHg/*μ*l)	0.653 ± 0.071	0.675 ± 0.098	0.835 ± 0.095^∗^	0.863 ± 0.094^#^
PRSW (mmHg/*μ*l)	68.16 ± 6.65	70.47 ± 6.75	84.12 ± 8.09^∗^	86.96 ± 9.70^#^
E*es* (mmHg/*μ*l)	0.75 ± 0.08	0.77 ± 0.09	0.94 ± 0.10^∗^	0.97 ± 0.10^#^

EF: ejection fraction; PAMP: preload adjusted maximal power; ESV: end-systolic volume; EDV: end-diastolic volume; E*max*: maximal elastance; ESPVR: end-systolic pressure-volume relationship; PRSW: preload recruitable stroke work; E*es*: end-systolic elastance. ^∗^ *p* < 0.05 versus EDC and GF; ^#^ *p* < 0.01 versus EDC and GF.

## Supplementary Materials

Supplementary Materials Supplement Figure 1: human mesenchymal stem cells (hMSCs) characteristics of cultured hMSCs were identified by a flow cytometric analysis of surface antigen expression. Representative images showed that most of hMSCs expressed both CD90 and CD105 antigens and did little express leukocyte common antigens CD45, or the hematopoietic lineage markers CD34 (*n* = 5/group). Supplement Figure 2: identification of hMSCs in collagen patch after 4-day cultivation. The collagen patch slices were immunohistochemically stained against CD105 antigen after 4 days of hMSC seeding. A large quantity of CD105-positive cells was identified on the surface or the shadow layer of scaffolds.
