# Supplementary material for: Improved Left Ventricular Aneurysm Repair with Cell- and Cytokine-Seeded Collagen Patches
Source: Stem Cells Int. 2018 Feb 13;2018:4717802. doi: 10.1155/2018/4717802 (PMC5831860; doi:10.1155/2018/4717802)
Supplement: Supplementary Materials — Supplement Figure 1: human mesenchymal stem cells (hMSCs) characteristics of cultured hMSCs were identified by a flow cytometric analysis of surface antigen expression. Representative images showed that most of hMSCs expressed both CD90 and CD105 antigens and did little express leukocyte common antigens CD45, or the hematopoietic lineage markers CD34 (n = 5/group). Supplement Figure 2: identification of hMSCs in collagen patch after 4-day cultivation. The collagen patch slices were immunohistochemically stained against CD105 antigen after 4 days of hMSC seeding. A large quantity of CD105-positive cells was identified on the surface or the shadow layer of scaffolds. [file 4717802.f1.pdf]

Supplementary Materials:

Abbreviation：

EDTA: Ethylene diaminetetraacetic acid

FITC: Fluorescein isothiocyanate

PBS: Phosphate buffer solution

PFA: Paraformaldehyde

OCT- Compound: Optimum Cutting Temperature Compound

HRP: Horse Reddish Peroxidase

DAB: Diaminobenzidine

Supplementary Methods

Identification of seeding cell with flow cytometry

To identify the phenotype of seeding cells, we performed flow cytometry to characterize the isolated cells (Passage 3) for the surface markers of CD90, CD105, CD45, and CD34. Briefly, the cultured cells were dissociated with 0.25% trypsin-EDTA and filtered through a 40-μm filter mesh and resuspended at the concentration of 1×10^5 cells/mL. After washed twice with PBS buffer, cells were incubated with anti-human CD45/FITC, anti-human CD90/FITC, anti-human CD105/FITC, anti-human CD34/FITC and isotype control at 4℃ for 30 minutes in the dark [1]. These monoclonal antibodies were obtained from Abcam company (Abcom-93758). Samples (n=5/group) were detected by the flow cytometer (Becton, Dickinson and Company, USA).

Immunohistochemistry:

0.5 x 10^6 cells were seeded into collagen scaffolds evenly and cultured in an incubator for 4 days as previously described [2]. Then, the constructs were fixed with 4% PFA for 24 hours and placed facing down in cryomolds with a thin layer of OCT compound (Tissue-Tek, 4583) at the bottom. The molds were filled up with OCT, snap-frozen with dry ice and cut into 5μm thick slices for staining. The staining procedure was performed as manufacture's protocol. Firstly, the sections were fixed in methanol prior to blocking with 10% horse serum for 5 minutes at 25°C. Then, 100μL of primary antibody (1:200, mouse monoclonal anti- human CD105 antigen, Abcom-11414) was incubated with the sample at 4℃ overnight, followed by the application of

100μL of HRP-conjugated secondary antibody (1:100, goat anti-mouse polyclonal antibody, Abcom-19195) for 1 hour at room temperature. Then, the slides were stained with DAB for 10 minutes, mounted, covered and imaged.

Supplementary Results

Seeding cells expressed surface markers of MSCs

The flow cytometry analysis showed that the average percentage of seeding cells that positively expressed CD90 and CD105 was 97.41% and 95.1% respectively, whereas less than 2% of the cell population expressed leukocyte common antigens, CD45 (1.83%), or the hematopoietic lineage markers CD34 (1.97%) (n=5/group). Our data identified that the isolated cells are conformed to the criteria of MSCs that International Society for Cellular Therapy proposed in 2006[3]. (Fig. Supp 1).

hMSCs survived after 4 days of in-vitro cultivation in scaffold

As our immunohistochemical staining illustrated, a large quantity of CD105 positive cells were identified in collagen patches 4 days after implanting. Further study showed that the positive cells were predominantly confined on the surface or the shadow layer of sponges, which might be caused by the devoid of dynamic stress of blood flow and the vascular vessels within sponge in static culturing condition.

Supplementary References

1. Wang X, Du Z, Liu X, Song Y, Zhang G, Wang Z, Wang Q, Gao Z, Wang Y and Wang W, Expression of CD44 standard form and variant isoforms in human bone marrow stromal cells,Saudi Pharm J. 2017;25(4):488-491

2. Chiu LL, Radisic M. Scaffolds with covalently immobilized VEGF and Angiopoietin-1 for vascularization of engineered tissues. Biomaterials. 2010;31(2):226-241.

3. Dominici M, Le Blanc K, Mueller I, Slaper-Cortenbach I, Marini F, Krause D, Deans R, Keating A, Prockop Dj, Horwitz E, Minimal criteria for defining multipotent mesenchymal stromal cells,The International Society for Cellular Therapy position statement. Cytotherapy. 2006;8(4):315-317.

Supplementary Figures

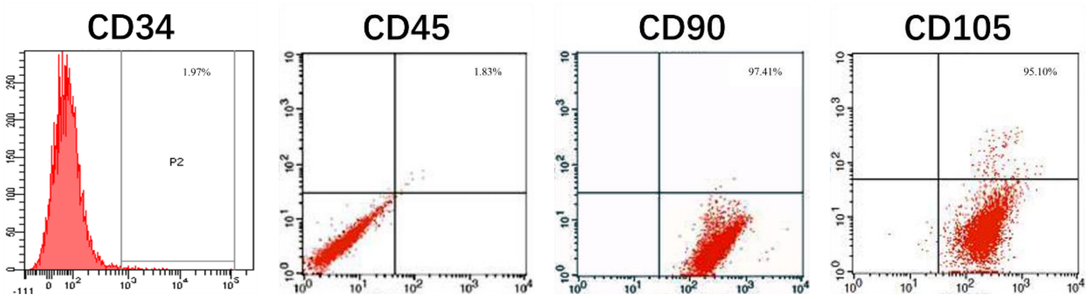

Supplement Figure 1: Identification of human mesenchymal stem cells (hMSCs)

Characteristics of cultured hMSCs were identified by a flow cytometric analysis of surface antigen expression. Representative images showed that most of hMSCs expressed both CD90, and CD105 antigens, and did little express leukocyte common antigens, CD45, or the hematopoietic lineage markers CD34 (n=5/group).

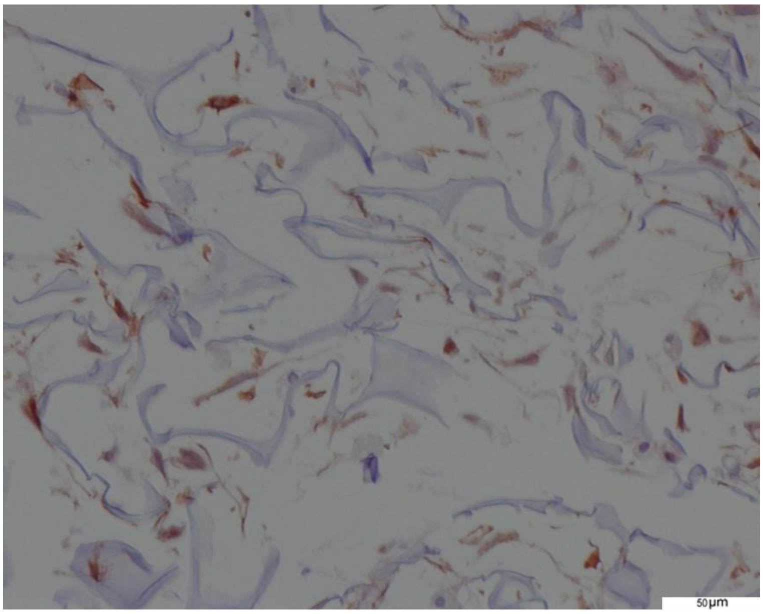

Supplement Figure 2: Identification of hMSCs in collagen patch after 4-day cultivation.

The collagen patch slices were immunohistochemically stained against CD105 antigen after 4 days of hMSCs seeding. A large quantity of CD105 positive cells were identified on the surface or the shadow layer of scaffolds.
